# Mid-Cretaceous carbon cycle perturbations and Oceanic Anoxic Events recorded in southern Tibet

**DOI:** 10.1038/srep39643

**Published:** 2016-12-21

**Authors:** Xiaolin Zhang, Kefan Chen, Dongping Hu, Jingeng Sha

**Affiliations:** 1 School of Earth and Space Sciences, University of Science and Technology of China, Hefei 230026, China; 2 LPS, Nanjing Institute of Geology and Palaeontology, Chinese Academy of Sciences, Nanjing 210008, China

## Abstract

The organic carbon isotope (δ^13^C_org_) curve for ~1.7-km-thick mid-Cretaceous strata of the Chaqiela section in Gamba area, southern Tibet is presented in this study. C-isotopic chemostratigraphic correlation combined with biostratigraphic constraints show that the Chaqiela section spans early Aptian through early Campanian period, and that almost all of the carbon cycle perturbations and Oceanic Anoxic Events during the mid-Cretaceous period are well recorded in the continental margin area of the southeastern Tethys Ocean. Significantly, two levels of methane-derived authigenic carbonates were identified at the onset of OAE1b near the Aptian-Albian boundary. We suggest that an increase in methane release from gas hydrates, potentially driven by sea-level fall and bottom water temperature increase, may have contributed to the large negative δ^13^C_org_ excursions and global warming during OAE1b.

The mid-Cretaceous period (125–80 Ma) witnessed a series of Oceanic Anoxic Events (OAEs) and dramatic climate changes, which are attributed to the intermittent global carbon cycle perturbations in ocean-atmosphere system^1^,^2^,^3^,^4^. The influx of CO_2_ and/or CH_4_ into the ocean and atmosphere has been considered to be the most important reason of the global warming and oceanic anoxia, whereas the widespread accumulation of organic carbon in marine deposits during the OAEs has been linked to the drawdown of atmospheric *p*CO_2_ and transient global cooling^1^,^4^,^5^,^6^. The carbon isotope excursions in carbonate and organic carbon had recorded these carbon cycle changes and can be faithfully used to mid-Cretaceous stratigraphic correlation^1^,^5^,^7^,^8^. A large number of mid-Cretaceous C-isotope curves have been reported during the past decades^3^,^7^,^8^,^9^,^10^,^11^,^12^,^13^. However, most studies were focused on the western Tethys, Atlantic, Western Interior Seaway, and Pacific, whereas only a few high resolution carbon isotope curves from eastern Tethys were presented^8^,^14^,^15^,^16^. In addition, no single section can provide a complete record of the entire succession of the mid-Cretaceous OAEs. Here we present a long-term δ^13^C_org_ record from mid-Cretaceous sediments in the Chaqiela section of Gamba area, southern Tibet.

The Chaqiela section (N28°13′7.1″; E88°37′45.3″) is situated about 13 km east from the Gamba County^17^,^18^. It is located at the southern subzone of Tethyan Himalayas on the northern margin of the Indian Plate and was located at the southern margin of the eastern Tethys during mid-Cretaceous period^14^,^19^,^20^. The Cretaceous strata of the Chaqiela section, mainly composed of shale, siltstone, marl, and limestone (Figs S1–S3), is subdivided into the Dongshan, Chaqiela, Gambacunkou, and Zongshan formations in ascending order (Fig. 1, Table S1)^17^,^19^,^21^,^22^,^23^,^24^,^25^,^26^. No obvious faults, folds, and hiatus were found in the Chaqiela section except that a short interval in the middle of the section was covered by gravels. Preliminary palaeontological studies show that the Cretaceous strata of the Chaqiela section extends from Aptian through Maastrichtian^17^,^18^,^21^. Hence, the Chaqiela section is perfect for studies on long-term Cretaceous carbon cycle variations and the oceanographic and climatic changes in eastern Tethys area.

## Results

**Figure 1:**
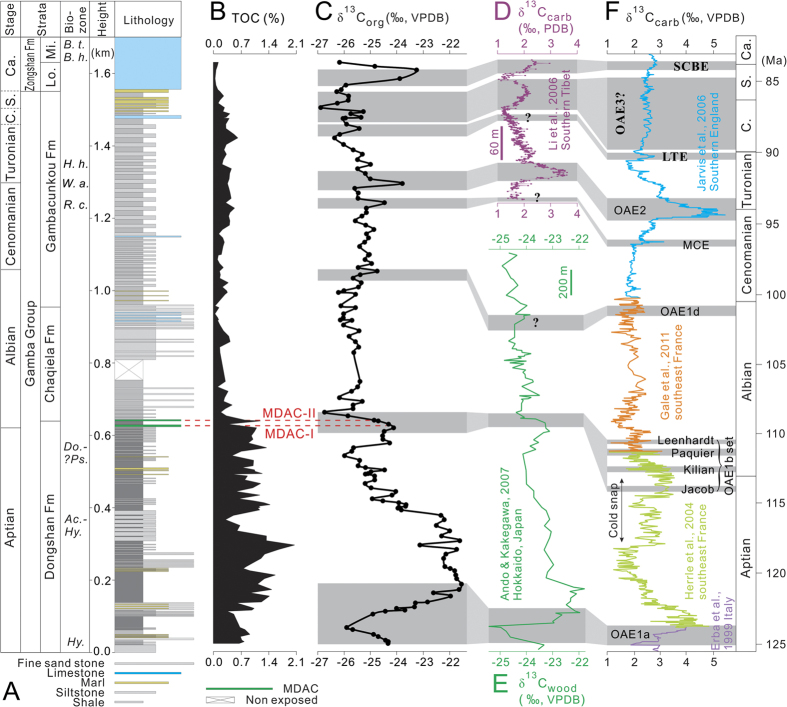
Carbon isotope records of the Chaqiela section and chemostratigraphic correlation. (**A**) Bio- and lithostratigraphy. (**B**) Total organic carbon (TOC, %). (**C**) Organic carbon isotopes. (**D**) Carbonate carbon isotope curve from the Gongzha section in Tingri area, southern Tibet^14^. (**E**) Wood carbon isotope curve from Hokkaido, Japan^37^. (**F**) Carbonate carbon isotope composite age-calibrated curve compiled from Erba *et al*.^10^, Herrle *et al*.^11^, Jarvis *et al*.^3^, and Gale *et al*.^12^. The composite carbon isotope record was tuned by Herrle *et al*.^13^ using the Gradstein *et al*.^5^ age concept. Red dotted lines show the position of two methane-derived authigenic carbonates (MDAC) beds. Gray areas represent correlative paleoceanographic events. OAE—Oceanic Anoxic Event; SCBE—Santonian/Campanian Boundary Event; LTE—Late Turonian Event; MCE—Middle Cenomanian Event; C.—Coniacian; S.—Santonian; Ca.—Campanian; Lo.—Lower; Mi.—Middle; *B. t*.—*Bournonia tibetica; B. h*.—*Bournonia haydeni; H. h*.—*Helvetoglobotruncana helvetica; W. a*.—*Whiteinella archaeocretacea; R. c*.—*Rotalipora cushmani; Do*.—*Douvilleiceras; ?Ps*.—*?Pseudosonneratia; Ac*.—*Acanthohoplites; Hy*.—*Hypacanthoplites*.

A total of 142 rock samples were collected from 1655 m-thick mid-Cretaceous strata in the Chaqiela section. The δ^13^C_org_ and total organic carbon (TOC) data for these rock samples were obtained (Table S2). The δ^13^C_org_ values show large fluctuations, range from −26.9‰ to −21.6‰ with an average of −24.8‰ (Fig. 1). The TOC values range from 0.03 to 2.05% (average 0.58%). The Dongshan Formation contains relatively high TOC content, ranging from 0.30% to 2.05% with an average of 0.96%, whereas the TOC values of Chaqiela, Gambacunkou, and Zongshan formations range from 0.03% to 0.60% with an average of 0.26%.

## Discussion

### Stratigraphic correlation and OAEs

The δ^13^C_org_ record of the Chaqiela section is marked by several negative and positive excursions of >1‰. Organic biomarker data indicate that the sedimentary organic matter in Gamba area was mainly derived from marine algae^27^,^28^, which precludes the possibility that the δ^13^C_org_ excursions were caused by the variation of organic matter sources. Based on the similar variation trends we correlated our δ^13^C_org_ curve in the Cheqiela section with the age-calibrated reference δ^13^C_carb_ curve in Europe compiled by Jarvis *et al*.^3^ and Herrle *et al*.^13^ (Fig. 1F). Almost all of mid-Cretaceous carbon cycle perturbations and OAEs can be identified in the Chaqiela section based on the carbon isotope chemostratigraphic correlation and biostratigraphic constraints.

The ammonite biostratigraphy for the Dongshan Formation at Chaqiela has been well studied^17^. Three ammonite assemblages/beds, i.e. *Hypacanthoplites* bed, *Hypacanthoplites*-*Acanthohoplites* lineage assemblage, and *Douvilleiceras-?Pseudosonneratia* assemblage, were established (Fig. 1A), indicating that the Dongshan Formation were deposited in Aptian-early Albian period^17^. Our C-isotope results show that the Dongshan Formation exhibits two prominent positive δ^13^C_org_ excursions. The first positive excursion, from −25.9‰ to −21.6‰, is located at the lower part of the Dongshan Formation and is preceded by a negative excursion from −24.4‰ to −25.9‰. This excursion feature corresponds well with the early Aptian OAE1a (Fig. 1F)^7^,^9^,^10^. The second positive excursion, from −26.0‰ to −24.3‰, is located at the uppermost of the Dongshan Formation. This positive δ^13^C_org_ excursion and the following relatively high value interval can be correlated to the late Aptian cold snap (Fig. 1F)^6^,^11^,^13^. A large negative δ^13^C_org_ excursion, from −24.1‰ to −26.7‰, at the boundary between the Dongshan Formation and the Chaqiela Formation follows this cold snap. It appears that this negative excursion corresponds to the OAE1b set interval (Jacob, Kilian, Paquier, and Leenhardt), which contain the Aptian-Albian boundary (Fig. 1F)^11^,^12^,^13^. Hence, our δ^13^C_org_ stratigraphy correlations suggest an Aptian-earliest Albian age for the Dongshan Formation, which are consistent with the ammonite biostratigraphic results. The Aptian-Albian boundary is placed at the onset of the negative δ^13^C_org_ shift (621 m), which is near the lithostratigraphic boundary between the Dongshan Formation and the Chaqiela Formation.

The large positive δ^13^C_org_ excursion in the middle part of the Gambacunkou Formation is identified as the latest Cenomanian OAE2 based on the regional and global C-isotope correlation and the foraminiferal stratigraphy in Gamba and Tingri area (Fig. 1, Fig. S4)^3^,^14^,^15^,^20^,^29^. Three foraminiferal zones, i.e. *Rotalipora cushmani* Zone, *Whiteinella archaeocretacea* Zone, and *Helvetoglobotruncana helvetica* Zone in ascending order, near the Cenomanian-Turonian (C-T) boundary have been established at Gamba and Tingri (Fig. S4)^14^,^15^,^20^,^29^. The large positive δ^13^C_org_ excursion within the three foraminifera zones at Gongzha were correlated well with the OAE2 intervals elsewhere^8^,^14^,^16^. The comparable variations in lithology, C-isotope, and foraminiferal biostratigraphy allow us to correlate OAE2 interval regionally among the Chaqiela, Zongshan, and Gongzha sections (Fig. S4). Hence, the large positive δ^13^C_org_ excursion in the middle part of the Gambacunkou Formation may represent the OAE2 interval near the C-T boundary. The minor positive δ^13^C_org_ excursion before OAE2 might correspond to the Middle Cenomanian Event (MCE).

The last large positive δ^13^C_org_ excursion, from −25.9‰ to −23.3‰, is located at the lower part of the Zongshan Formation (Fig. 1C). The limestone of the Zongshan Formation was shown to have deposited during Campanian-Maastrichtian period based on the biostratigraphic data (Table S1)^19^,^21^,^23^,^24^,^26^. Two rudist species, i.e. *Bournonia haydeni* Douville and *Bournonia tibetica* Douville, were identified in the middle and upper part of the Zongshan Formation (Fig. 1A)^18^, indicating the Campanian-Maastrichtian in age. Therefore, the δ^13^C_org_ curves that can be correlated with that from the Tingri area (Fig. 1D) and southern England (Fig. 1F) suggest that the last large positive δ^13^C_org_ excursion in our study may correspond to the Santonian-Campanian Boundary Event (SCBE)^3^,^8^,^14^,^16^, which is at the lithostratigraphic boundary between the Gambacunkou Formation and Zongshan Formation. The amplitude of the positive δ^13^C_org_ excursion of SCBE in the Cheqiela section (2.6‰) is far larger than the δ^13^C_carb_ records in the English Chalk sections (~0.5‰). The large amplitude shift of δ^13^C during SCBE is also recorded in skeletal calcite of mollusk in southwestern British Columbia, Canada^30^. The findings of large amplitude both in Tethys Ocean and northeastern Pacific suggest that SCB positive C-isotope shift may reflect a major perturbation of the global carbon cycle.

There are also a few minor positive δ^13^C_org_ excursions in the Chaqiela section. The one located at the lowermost part of the Gambacunkou Formation, from −25.7‰ to −24.7‰, may correspond to the OAE1d interval in the latest Albian (Fig. 1F), which is consistent with the previous biostratigrapic studies in Gamba area^19^. The three minor positive δ^13^C_org_ excursions at the uppermost part of the Gambacunkou Formation are comparable with the latest Turonian-Santonian δ^13^C_carb_ curve from southern England despite some differences in amplitude for later two positive excursions (Fig. 1F)^3^. The first positive excursion may correspond to the late Turonian Events (LTE), which can be used to define the Turonian-Coniacian boundary in the Chaqiela section. The amplitude of the following two positive δ^13^C_org_ excursions in Coniacian-Santonian period is larger than the δ^13^C_carb_ records in the English Chalk sections. The relatively large excursion amplitude is consistent with the previous δ^13^C_carb_ records from Tingri area of South Tibet (Fig. 1D)^14^,^16^ and supports the view that Coniation-Santonian positive C-isotope shifts reflect a major perturbation of the global carbon cycle related to OAE3 (ref. 16) though the organic-rich sediments during this period mainly restricted to the Atlantic Ocean and adjacent basins^31^.

### Methane release during OAE1b

**Figure 2:**
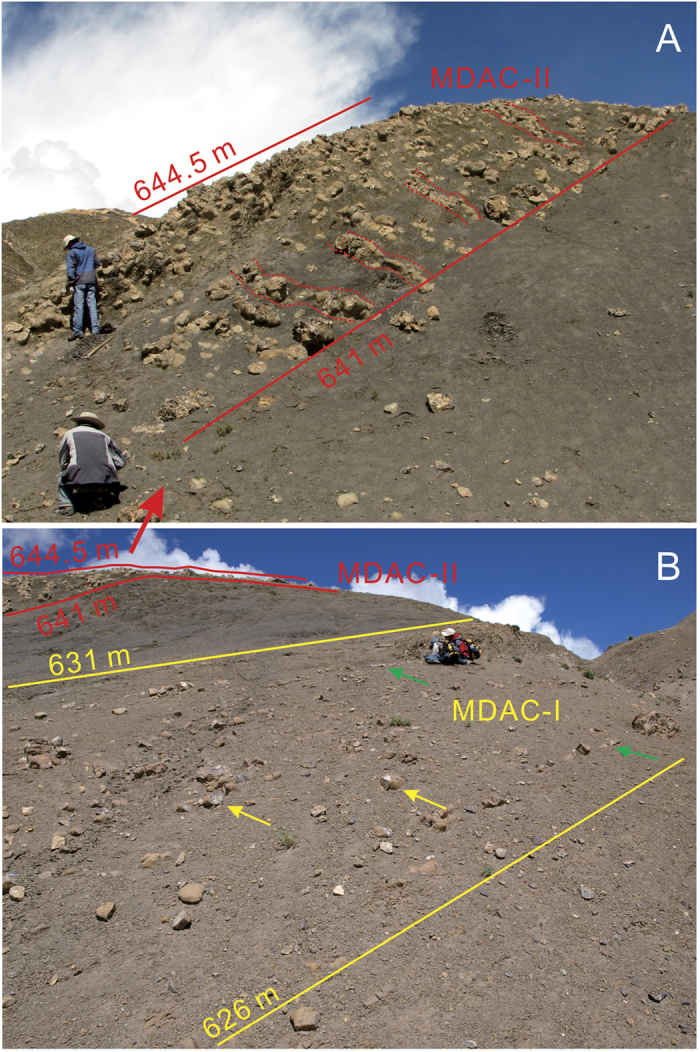
Photos of methane-derived authigenic carbonates (MDACs). (**A**) MDAC-II. Red dotted lines show tubular concretions elongated at high angles to the bedding. (**B**) MDAC-I. Yellow arrows show tubular conduits parallel to the bedding. Green arrows show tabular concretions.

The release of ^13^C-depleted methane from seabed gas hydrates was proposed to be the most likely cause of the large negative δ^13^C shift in both δ^13^C_carb_ and δ^13^C_org_ during OAE1b interval^32^,^33^. However, the emplacement of the Kerguelen LIP and/or the decrease of the organic carbon burial rate were also proposed to be the possible causes^2^,^6^,^34^. In this study we identified that two levels of methane-derived authigenic carbonates (MDAC) coincide with the onset of OAE1b large negative δ^13^C_org_ excursion (2.6‰) in the Chaqiela section (Figs 1 and 2). These MDACs are widespread in Gamba area^35^. The δ^13^C values ranging between −27.37‰ and −23.85‰ suggest contribution of the methane release from hydrate to the formation of these MDACs^35^. Hence, our results provide strong evidence for a causal link between methane release from gas hydrate and the large negative δ^13^C_org_ excursion during OAE1b interval. The two MDAC beds identified in the Chaqiela section have different morphological characters and they are with different δ^13^C excursions in magnitude. The MDAC-I (Figs 1 and 2B), from 626 m to 631 m, is composed of relatively small size of tubular concretions (conduits) and thin tabular concretions parallel to the bedding and is followed by a negative δ^13^C_org_ shift of 0.8‰ (from −24.1‰ to −24.9‰). The MDAC-II (Figs 1 and 2A), from 641 m to 644.5 m, comprises well-developed relatively large tubular concretions elongated at high angles to the bedding. This feature indicates vertically directed flow of methane-rich fluids and methane release, producing a large negative δ^13^C_org_ shift of 1.8‰ (from −24.9‰ to −26.7‰). The MDACs were also found underneath and within the OAE1b black shale level in southern France^36^, suggesting that the methane release may be of global significance. Hence, our results provide strong evidence for that methane release from gas hydrate may have played an important role in the large negative δ^13^C excursion in both δ^13^C_carb_ and δ^13^C_org_ during OAE1b interval.

The negative δ^13^C anomaly during OAE1b was also documented in terrestrial wood fragments (Fig. 1E)^37^, leaf wax *n*-alkane^33^, and was accompanied by a synchronous sudden global warming^6^,^33^. The terrestrial isotopic record suggest that the methane released from gas hydrate passed throughout the water column and reached the atmosphere. However, the relatively large δ^13^C_org_ excursion in the Chaqiela section (2.6‰), compare to the terrestrial plant records (~1–1.5‰), implies that the majority of methane released in continental margin area of the southeastern Tethys Ocean was dissolved in the water column. Only part of methane entered into the atmosphere and influenced global carbon cycle and climate.

Gas hydrate dissociation can result from pressure decrease due to sea-level fall or temperature increase of bottom water^38^,^39^. A prominent global sea level fall coincides with the sudden negative δ^13^C excursion of OAE1b during Aptian-Albian transition period^40^,^41^,^42^. Therefore, the reduction in hydrostatic pressure caused by sea-level fall may have triggered hydrate dissociation in sediments and massive methane release during OAE1b. In addition, the drifting of the Indian Plate northwards and the changes in ocean circulation may have resulted in the increase of bottom water temperature and contributed to the hydrate dissociation in continental margin area of the southeastern Tethys Ocean. In contrast, the cold snap before OAE1b and the high sea level benefitted the gas hydrate stability. Furthermore, high TOC values of the Dongshan Formation indicate that the organic rich sediments below the OAE1b MDAC levels could be the source of the CH_4_ generation and the formation of gas hydrate.

## Methods

The rock samples were ground to powder with a mortar and pestle and then weighted and decarbonated with 10% HCl to remove all inorganic carbon. The samples were subsequently washed several times with deionized water to remove the HCl traces and then dried in an oven at 50 °C. Total organic carbon (TOC) and δ^13^C_org_ were analyzed on a ThermoFinnigan Flash Elemental Analyzer coupled in continuous flow to a ThermoFinnigan MAT 253 Mass Spectrometer. The δ^13^C_org_ values are expressed in per mil (‰) relative to VPDB standard.

## Additional Information

**How to cite this article**: Zhang, X. *et al*. Mid-Cretaceous carbon cycle perturbations and Oceanic Anoxic Events recorded in southern Tibet. *Sci. Rep.* **6**, 39643; doi: 10.1038/srep39643 (2016).

**Publisher's note:** Springer Nature remains neutral with regard to jurisdictional claims in published maps and institutional affiliations.

## Supplementary Material

Supplementary Information
