# Supplementary material for: Mid-Cretaceous carbon cycle perturbations and Oceanic Anoxic Events recorded in southern Tibet
Source: Sci Rep. 2016 Dec 21;6:39643. doi: 10.1038/srep39643 (PMC5175164; doi:10.1038/srep39643)
Supplement: Supplementary Information [file srep39643-s1.pdf]

Supplementary Information

Mid-Cretaceous carbon cycle perturbations and Oceanic Anoxic Events recorded in southern Tibet

Xiaolin Zhang[1*], Kefan Chen[1], Dongping Hu[1], Jingeng Sha[2]

[1]School of Earth and Space Sciences, University of Science and Technology of China, Hefei 230026, China.

[2]LPS, Nanjing Institute of Geology and Palaeontology, Chinese Academy of Sciences, Nanjing 210008, China.

*Corresponding author: XZ (zhxl2012@ustc.edu.cn)

Figure legends

Fig. S1: Lithology of the Dongshan Formation in the Chaqiela section. (A) Fine sandstone intercalated with siltstone. (B) & (C) Black shale.

Fig. S2: Lithology of the Chaqiela Formation in the Chaqiela section. (A) Siltstone intercalated with shale. (B) Siltstone.

Fig. S3: Lithology of the Gambacunkou Formation and Zongshan Formation in the Chaqiela section. (A) Grey shale intercalated with thin-bedded siltstone in the Gambacunkou Formation. (B) Marl intercalated with grey shale in the Gambacunkou Formation. (C) Marl in the Gambacunkou Formation and limestone in the Zongshan Formation with the boundary at 1556 m.

Fig. S4: Stratigraphic correlation of the Cretaceous strata in Gamba and Tingri area, southern Tibet. Red dashed lines show the lithostratigraphic correlation between the Chaqiela section and the Zongshan section in Gamba area. The Zongshan section and the Chaqiela section are only ~12 km apart from each other. The lithologic features can be easily correlated between these two sections due to the similar sedimentary environment. Based on the similar position in lithologic units and the similar excursion trends we correlated the large positive carbon isotope excursion in the middle part of the Gambacunkou Formation in the Chaqiela section with the OAE2 interval (pink shaded area) in the Zongshan section. Three foraminifera zones were

established, i.e. *Rotalipora cushmani* Zone, *Whiteinella archaeocretacea* Zone, and *Helvetoglobotruncana helvetica* Zone in ascending order, near Cenomanian-Turonian boundary in both the Zongshan section (Gamba area) and the Gongzha section (Tingri area). Hence, we can further correlate the OAE2 interval in Gamba area with the Tingri area (pink shaded area). In addition, the large positive carbon isotope excursion near the boundary between the Gambacunkou Formation and the Zongshan Formation can be correlated with each other among the Chaqiela section, Zongshan section, and Gongzha section (green shaded area).

Fig. S1

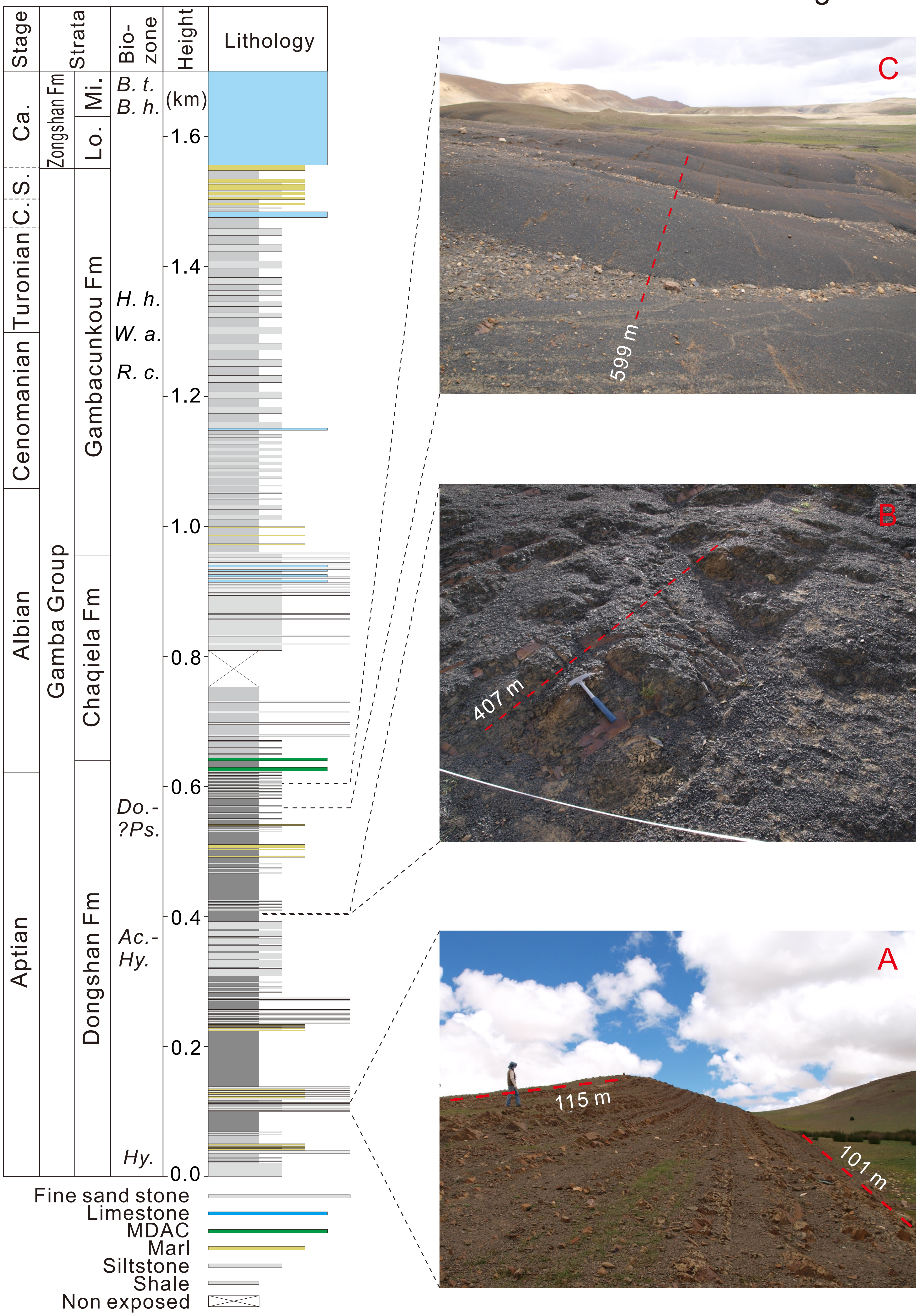

Stage
Strata
Bio-zone
Height
Lithology
Ca.
S.
C.
Turonian
Cenomanian
Albian
Aptian
Zongshan Fm
Gamba Group
Mi.
Lo.
Gambacunkou Fm
Chaqiela Fm
Dongshan Fm
B. t.
B. h.
H. h.
W. a.
R. c.
Do.-
?Ps.
Ac.-
Hy.
Hy.
(km)
1.6
1.4
1.2
1.0
0.8
0.6
0.4
0.2
0.0
Fine sand stone
Limestone
MDAC
Marl
Siltstone
Shale
Non exposed
C
599 m
B
407 m
A
115 m
101 m

Fig. S2

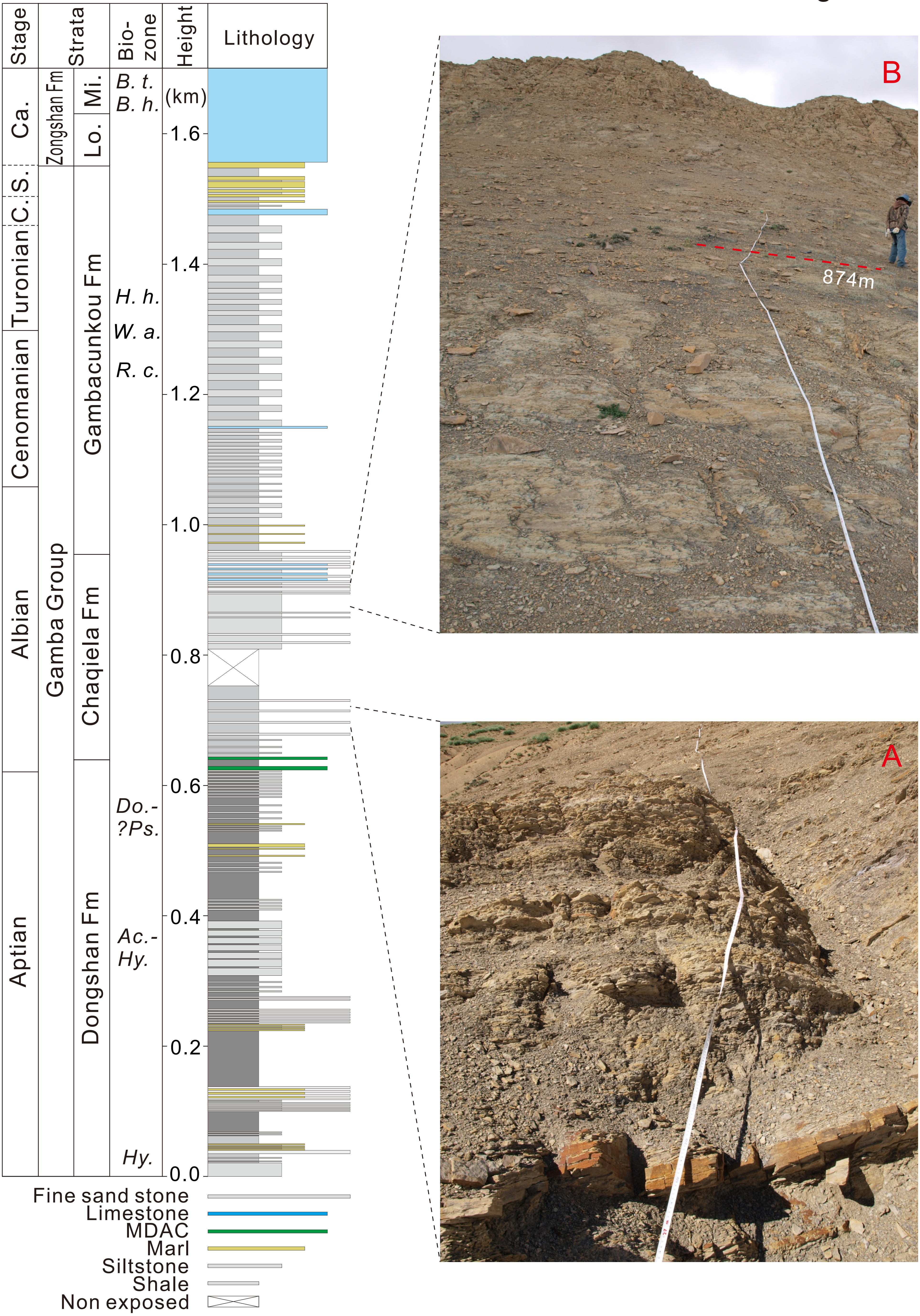

Stage
Strata
Bio-zone
Height
Lithology
Ca.
S.
C.
Turonian
Cenomanian
Albian
Aptian
Zongshan Fm
Gamba Group
Mi.
Lo.
Gambacunkou Fm
Chaqiela Fm
Dongshan Fm
B. t.
B. h.
H. h.
W. a.
R. c.
Do.-
?Ps.
Ac.-
Hy.
Hy.
(km)
1.6
1.4
1.2
1.0
0.8
0.6
0.4
0.2
0.0
Fine sand stone
Limestone
MDAC
Marl
Siltstone
Shale
Non exposed
B
874m
A

Fig. S3

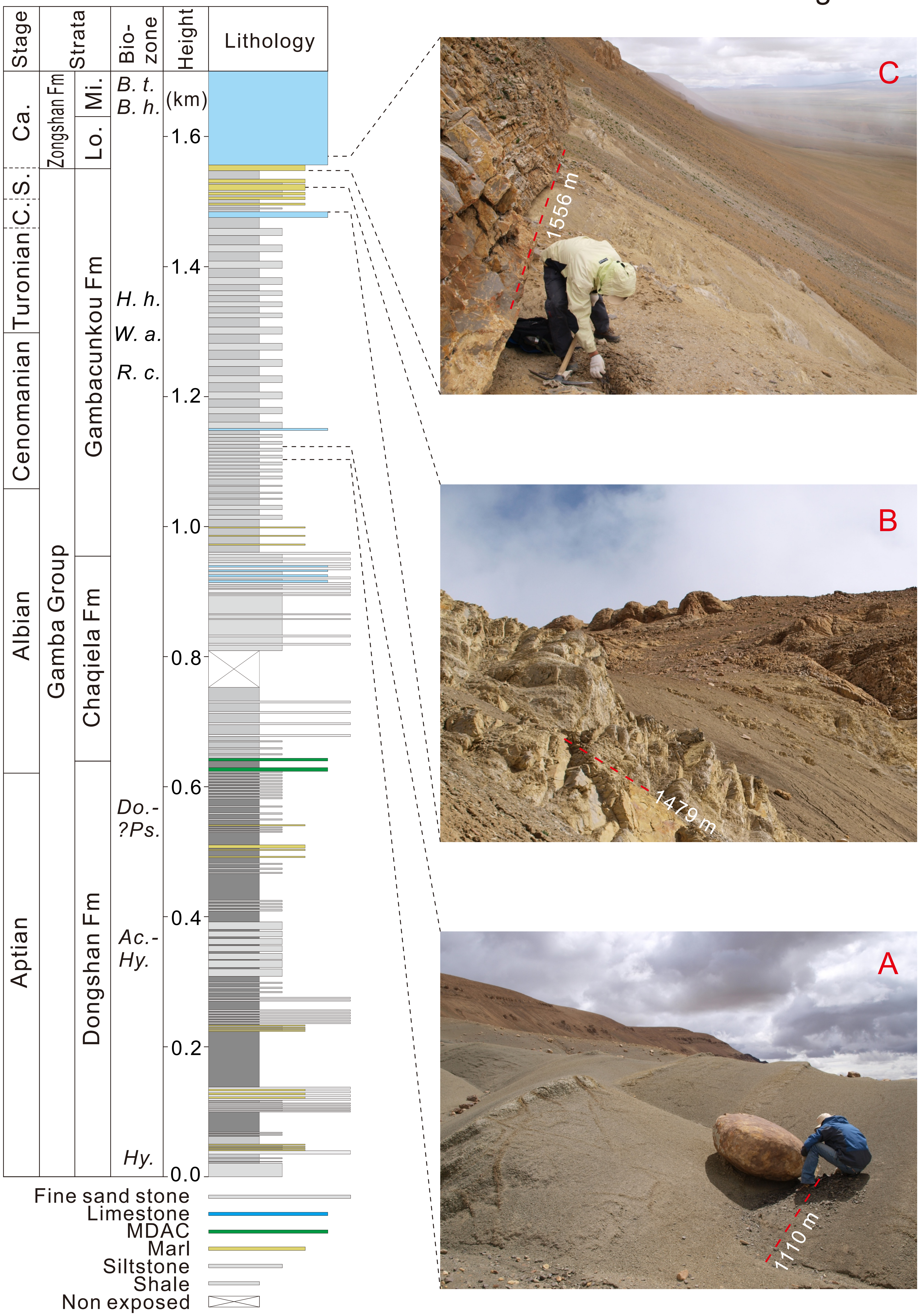

Stage
Strata
Bio-zone
Height
Lithology
Ca.
S.
C.
Turonian
Cenomanian
Albian
Aptian
Zongshan Fm
Mi.
Lo.
Gamba Group
Gambacunkou Fm
Chaqiela Fm
Dongshan Fm
B. t.
B. h.
H. h.
W. a.
R. c.
Do.-?Ps.
Ac.-Hy.
Hy.
(km)
1.6
1.4
1.2
1.0
0.8
0.6
0.4
0.2
0.0
Fine sand stone
Limestone
MDAC
Marl
Siltstone
Shale
Non exposed
C
1556 m
B
1479 m
A
1110 m

Fig. S4

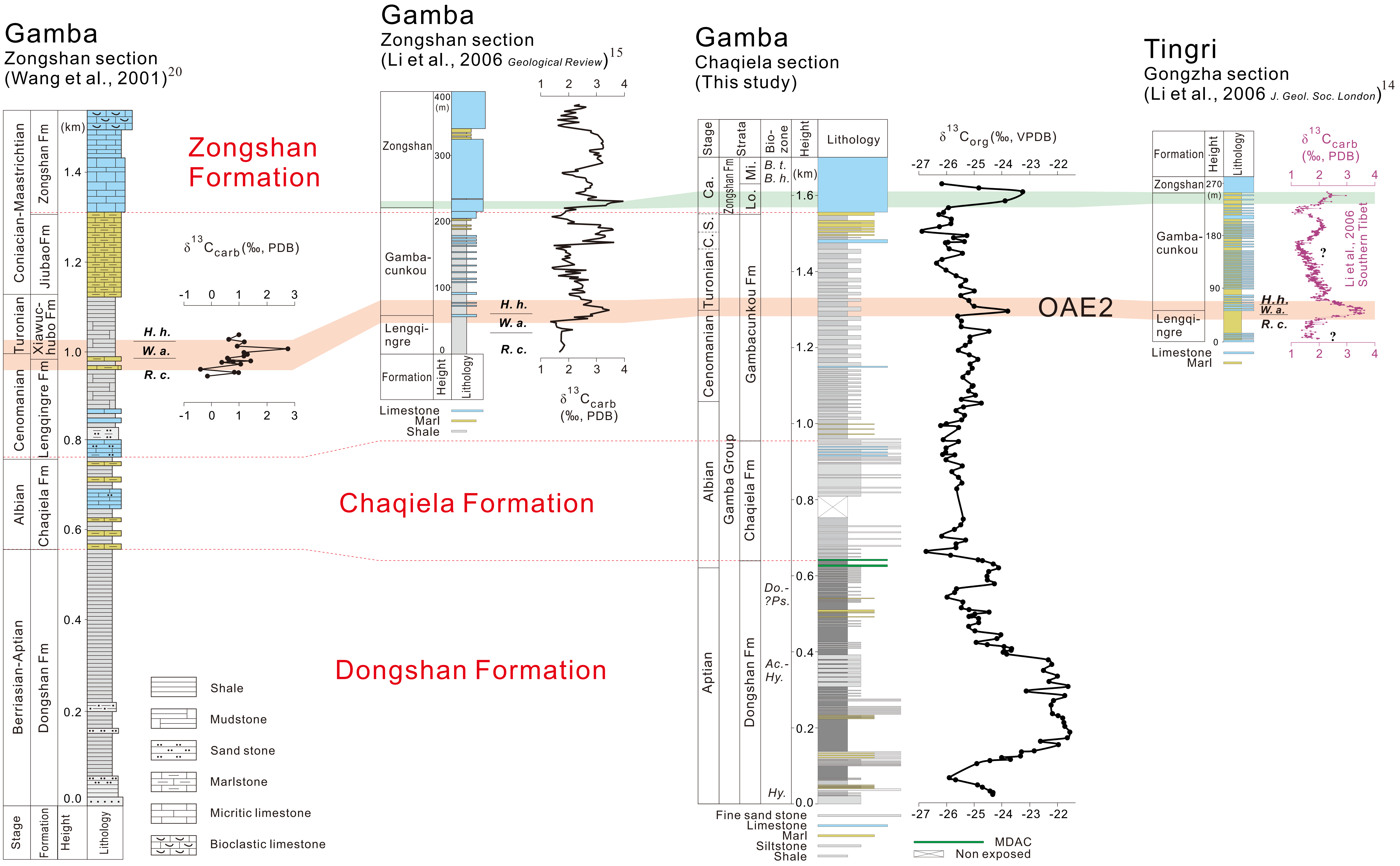

Gamba
Zongshan section
(Wang et al., 2001)20
Gamba
Zongshan section
(Li et al., 2006 Geological Review)15
Gamba
Chaqiela section
(This study)
Tingri
Gongzha section
(Li et al., 2006 J. Geol. Soc. London)14
Zongshan Formation
Chaqiela Formation
Dongshan Formation
OAE2
δ13Ccarb (‰, PDB)
δ13Corg (‰, VPDB)
Shale
Mudstone
Sand stone
Marlstone
Micritic limestone
Bioclastic limestone
Limestone
Marl
Shale
Fine sand stone
Siltstone
MDAC
Non exposed
Stage
Formation
Height
Lithology
Strata
Bio-zone
Coniacian-Maastrichtian
Turonian
Cenomanian
Albian
Berriasian-Aptian
Zongshan Fm
JiubaoFm
Xiawuc-hubo Fm
Lengqingre Fm
Chaqiela Fm
Dongshan Fm
Gamba Group
Gambacunkou Fm
Gamba-cunkou
Lengqi-ngre
Zongshan
H. h.
W. a.
R. c.
Do.-?Ps.
Ac.-Hy.
Hy.
B. t.
B. h.
Li et al., 2006
Southern Tibet

Table S1: Subdivision of Cretaceous strata in Gamba area, southern Tibet

Stage	(21) Wen et al., 1974	(22) Wang et al., 1980	(23) Wan et al., 1985 (19) Liu & Einsele, 1994	(24) Wan et al., 2000	(25) Yue et al., 2006	(26) Xiong et al., 2010	(17) Peng et al.,2014 This study		
Maastrichtian									
Campanian	Zongshan Fm		Zongshan Fm	Zongshan Fm		Zongshan Fm	Zongshan Fm	Dominated by limestone	~200m
Santonian			Jiubao Fm						
Coniacian				Gambacunkou Fm					
Turonian		Gambacunkou Fm	Xiawuchubo Fm			Gambacunkou Fm	Gambacunkou Fm	Grey shale intercalated with siltstone and marl	598m
Cenomanian			Lengqingre Fm	Lengqingre Fm					
Albian	Gamba Group	Chaqiela Fm	Chaqiela Fm	Chaqiela Fm	Chaqiela Fm	Chaqiela Fm	Chaqiela Fm	Grey shale and siltstone intercalated with fine sandstone and limestone	318m
Aptian					Dongshan Fm	Dongshan Fm	Dongshan Fm	Black shale intercalated with siltstone, fine sand stone, and marl	640m
Barremian		Dongshan Fm	Dongshan Fm	Dongshan Fm				↓ ?	
Hauterivian									
Valanginian					Jilu Group	Gucuo Fm			
Berriasian									

Table S2: Organic carbon isotopic data of the Chaqiela section

Sample	Depth (m)	$\delta^{13}C_{org}$	TOC (%)	Sample	Depth (m)	$\delta^{13}C_{org}$	TOC (%)
CQL-150	1631.0	-26.17	0.12	CQL-77	704.0	-26.17	0.28
CQL-149	1620.0	-24.84	0.09	CQL-76	694.0	-25.31	0.25
CQL-148	1610.0	-23.26	0.06	CQL-75	684.0	-25.66	0.47
CQL-147	1586.0	-23.89	0.04	CQL-74	674.0	-25.66	0.29
CQL-145	1568.0	-25.93	0.06	CQL-73	664.0	-26.74	0.18
CQL-143	1555.5	-26.11	0.10	CQL-72	654.0	-25.85	0.32
CQL-142	1550.0	-26.28	0.16	CQL-71	642.0	-24.86	0.80
CQL-141	1538.0	-25.83	0.25	CQL-70	640.0	-24.71	1.18
CQL-140	1526.0	-25.84	0.15	CQL-69	631.0	-24.32	0.36
CQL-139	1518.0	-26.25	0.11	CQL-68	621.0	-24.12	1.08
CQL-138	1506.0	-26.88	0.27	CQL-67	611.0	-24.48	1.03
CQL-137	1495.0	-25.27	0.24	CQL-66	599.0	-24.54	0.95
CQL-136	1489.0	-25.75	0.41	CQL-65	589.0	-24.53	0.99
CQL-135	1479.0	-25.36	0.31	CQL-64	579.0	-24.27	0.87
CQL-134	1477.0	-25.95	0.21	CQL-63	566.0	-25.64	1.34
CQL-133	1476.0	-26.04	0.39	CQL-62	556.0	-25.71	0.70
CQL-132	1460.0	-25.90	0.31	CQL-61	544.0	-25.98	0.46
CQL-131	1448.0	-25.43	0.31	CQL-60	531.0	-25.41	1.01
CQL-130	1432.0	-26.16	0.52	CQL-58	516.0	-25.47	1.00
CQL-129	1422.0	-26.36	0.61	CQL-57	511.0	-25.19	0.72
CQL-128	1404.0	-26.02	0.32	CQL-56	504.5	-24.47	1.03
CQL-127	1389.0	-25.65	0.26	CQL-55	499.5	-24.98	1.05
CQL-126	1377.0	-25.31	0.32	CQL-54	494.5	-25.09	1.23
CQL-125	1362.0	-25.49	0.24	CQL-52	493.0	-25.19	0.43
CQL-124	1349.0	-25.00	0.21	CQL-51	488.0	-24.85	0.85
CQL-123	1337.0	-25.49	0.45	CQL-50	477.0	-24.84	0.79
CQL-122	1324.0	-25.20	0.40	CQL-49	467.0	-25.20	1.19
CQL-121	1309.0	-25.01	0.39	CQL-48	455.0	-24.98	0.99
CQL-120	1296.0	-23.80	0.35	CQL-47	445.0	-24.04	0.56
CQL-119	1283.0	-25.60	0.38	CQL-46	435.0	-24.19	1.17
CQL-118	1270.0	-25.46	0.42	CQL-45	425.0	-24.93	0.63
CQL-117	1255.0	-25.47	0.44	CQL-44	419.0	-24.53	1.01
CQL-116	1243.0	-24.47	0.30	CQL-43	414.0	-23.93	1.27
CQL-115	1228.0	-25.16	0.53	CQL-42	408.5	-23.66	1.43
CQL-114	1216.0	-25.15	0.41	CQL-41	404.0	-23.67	1.12
CQL-113	1206.0	-25.33	0.38	CQL-40	401.5	-23.93	1.17
CQL-112	1193.0	-25.57	0.41	CQL-39	399.0	-23.96	1.11
CQL-111	1181.0	-25.18	0.40	CQL-38	394.0	-23.83	1.16
CQL-110	1169.0	-24.87	0.22	CQL-36	379.0	-22.34	0.50
CQL-109	1157.0	-25.15	0.13	CQL-35	367.0	-22.21	0.30
CQL-108	1146.0	-25.07	0.23	CQL-34	351.0	-22.51	0.66
CQL-107	1134.0	-25.22	0.27	CQL-33	336.0	-22.01	0.59
CQL-106	1122.0	-25.41	0.24	CQL-32	322.0	-22.31	1.33
CQL-105	1100.0	-25.04	0.20	CQL-31	309.0	-21.63	1.33
CQL-104	1098.0	-25.08	0.12	CQL-30	297.0	-23.13	2.05
CQL-103	1086.0	-25.21	0.10	CQL-29	285.0	-21.74	1.65
CQL-102	1075.0	-24.96	0.15	CQL-28	272.0	-22.15	1.29
CQL-101	1064.0	-25.46	0.24	CQL-27	260.0	-22.23	1.20
CQL-100	1054.0	-24.75	0.15	CQL-26	237.5	-22.19	0.82
CQL-99	1044.0	-25.39	0.17	CQL-24	232.0	-21.98	0.75
CQL-98	1034.5	-25.66	0.15	CQL-23	226.0	-21.82	0.91
CQL-97	1022.0	-25.35	0.37	CQL-22	214.0	-21.78	1.56
CQL-96	1010.0	-25.45	0.22	CQL-21	204.0	-21.74	1.28
CQL-95	1000.0	-26.00	0.32	CQL-20	189.0	-21.56	1.11
CQL-94	995.0	-26.21	0.35	CQL-19	174.0	-21.65	1.47
CQL-93	990.0	-25.56	0.29	CQL-18	165.0	-22.62	0.92
CQL-92	958.0	-26.12	0.04	CQL-17	156.0	-21.97	1.41
CQL-91	953.0	-25.57	0.13	CQL-16	139.5	-22.84	1.20
CQL-90	936.0	-26.02	0.03	CQL-15	137.0	-23.30	0.58
CQL-89	925.0	-26.03	0.10	CQL-14	126.0	-23.34	0.91
CQL-88	919.0	-26.14	0.13	CQL-13	122.0	-24.02	1.02
CQL-87	918.0	-25.70	0.10	CQL-12	117.0	-23.70	0.97
CQL-86	905.0	-26.02	0.16	CQL-11	114.0	-24.44	0.34
CQL-85	889.0	-25.43	0.25	CQL-10	106.0	-24.91	0.51
CQL-84	874.0	-25.81	0.38	CQL-9	69.0	-25.90	0.66
CQL-83	859.0	-25.57	0.42	CQL-8	63.5	-25.67	0.79
CQL-82	844.0	-25.45	0.15	CQL-7	50.0	-24.89	0.91
CQL-81	829.0	-25.63	0.16	CQL-5	44.0	-24.71	0.68
CQL-80	749.0	-25.40	0.30	CQL-3	34.0	-24.43	0.71
CQL-79	734.0	-25.49	0.10	CQL-2	30.0	-24.33	0.77
CQL-78	722.0	-25.72	0.37	CQL-1	25.0	-24.34	0.65
